# HMB and Liraglutide Confer Complementary Protection Against Lipotoxic and Atrophic Alterations in High-Glucose Plus Free Fatty Acid-Treated C2C12 Myotubes

**DOI:** 10.3390/nu18121865

**Published:** 2026-06-09

**Authors:** Li-Yuan Chen, Shao-Hsing Weng, Hsin-Hua Li, Chen-Hsing Su, Sing-Hua Tsou, Kuei-Chuan Chan, Chien-Ning Huang, Hui-Chih Hung, Sheng-Chieh Lin, Chih-Li Lin

**Affiliations:** 1 Institute of Medicine, Chung Shan Medical University, Taichung 402, Taiwan; cato@csmu.edu.tw (L.-Y.C.); kukuking0215@gmail.com (S.-H.W.); cshy049@csmu.edu.tw (C.-N.H.); 2 Department of Physical Therapy, Chung Shan Medical University, Taichung 402, Taiwan; 3 Department of Exercise Health Science, National Taiwan University of Sport, Taichung 404, Taiwan; vivid529@hotmail.com; 4 Department of Neurosurgery, Chung Shan Medical University Hospital, Taichung 402, Taiwan; jason910274@gmail.com; 5 Department of Medical Research, Chung Shan Medical University Hospital, Taichung 402, Taiwan; zinminid@gmail.com; 6 Department of Internal Medicine, Chung Shan Medical University Hospital, Taichung 402, Taiwan; chenkuei@ms16.hinet.net; 7 School of Medicine, Chung Shan Medical University, Taichung 402, Taiwan; 8 Department of Life Sciences, National Chung Hsing University, Taichung 402, Taiwan; hchung@dragon.nchu.edu.tw; 9 Department of Orthopedics, Chung Shan Medical University Hospital, Taichung 402, Taiwan

**Keywords:** sarcopenia, β-hydroxy-β-methylbutyrate (HMB), liraglutide, type 2 diabetes (T2D), mTOR signaling

## Abstract

Background/Objectives: Type 2 diabetes (T2D)-associated sarcopenia is characterized by impaired insulin signaling, lipotoxicity, oxidative stress, and progressive muscle loss. Although liraglutide improves glucose control and reduces lipid burden, its ability to preserve muscle integrity under diabetic lipotoxic conditions remains limited. This study investigated whether β-hydroxy-β-methylbutyrate (HMB) could enhance liraglutide-mediated protection against high-glucose plus free fatty acid (HG+FFA)-induced injury in skeletal muscle cells. Methods: Differentiated C2C12 myotubes were exposed to HG+FFA to establish a sublethal lipotoxic model and treated with liraglutide, HMB, or their combination. Cell viability, lipid accumulation, myotube morphology, insulin signaling, glucose uptake, mitochondrial function, reactive oxygen species (ROS), antioxidant gene expression, and atrophy-related signaling were assessed. Results: HG+FFA induced marked lipid droplet accumulation, impaired insulin signaling, reduced glucose uptake, disrupted mitochondrial membrane potential, increased ROS production, suppressed antioxidant gene expression, and promoted an atrophic phenotype characterized by increased atrogin-1 and MuRF1 and reduced myogenic markers. Liraglutide alone reduced large lipid droplets and partially improved insulin signaling but showed limited efficacy in preserving the myotube phenotype. HMB alone exerted modest effects on lipid accumulation but preserved myotube area. Notably, combined HMB and liraglutide treatment more effectively reduced lipid burden, restored insulin signaling and glucose uptake, attenuated mitochondrial dysfunction and oxidative stress, restored antioxidant gene expression, and preserved MyHC-positive area and myotube diameter while suppressing atrogin-1/MuRF1 activation. These protective effects were largely attenuated by rapamycin, indicating at least partial dependence on mTOR-associated signaling. Conclusions: Overall, HMB and liraglutide exert complementary protective effects against diabetic lipotoxic and atrophic stress, supporting the potential utility of this combination strategy for T2D-associated sarcopenia.

## 1. Introduction

Type 2 diabetes (T2D) is a chronic metabolic disorder characterized by insulin resistance and impaired insulin secretion, leading to persistent hyperglycemia. It is associated with a range of complications, including cardiovascular disease, neuropathy, and retinopathy, all of which significantly impact quality of life and reduce life expectancy. One of the less recognized but increasingly prevalent complications of T2D is sarcopenia, a condition defined by the progressive loss of muscle mass and strength. Sarcopenia in T2D patients is particularly concerning, as it not only contributes to physical disability and frailty but also worsens the overall prognosis of T2D, creating a vicious cycle of deteriorating metabolic and muscular health [1]. The development of sarcopenia in T2D is driven by several factors, primarily metabolic dysregulation, chronic low-grade inflammation, and oxidative stress. Elevated blood glucose and insulin resistance interfere with muscle protein synthesis and promote protein degradation, contributing to muscle atrophy [2]. Additionally, increased levels of free fatty acids (FFAs) are known to induce lipotoxicity, where excess lipids accumulate in muscle cells as lipid droplets [3]. This accumulation disrupts cellular functions, impairs muscle regeneration, and exacerbates muscle damage. Studies have shown that lipotoxicity, driven by both elevated glucose and FFA levels, accelerates muscle wasting and impairs the regenerative capacity of muscle fibers, leading to further dysfunction [4]. This metabolic and lipotoxic cascade perpetuates muscle wasting, complicating T2D and sarcopenia management. Moreover, the activation of catabolic signaling pathways, such as the ubiquitin–proteasome system, is a hallmark of muscle wasting in T2D [5]. This pathway is regulated by key muscle-specific atrophy markers like atrogin-1 and muscle ring finger-1 (MuRF1), which mediate the breakdown of muscle proteins in response to metabolic stress [6]. The increased expression of these markers in T2D patients underscores their role in muscle wasting and highlights them as potential therapeutic targets. In addition, markers of muscle differentiation, such as myogenin, myogenic differentiation factor (MyoD), and myosin heavy chain (MyHC), are critical for maintaining muscle integrity. Myogenin and MyoD are essential transcription factors involved in muscle cell differentiation, while MyHC is a structural protein that plays a central role in muscle contractility. Alterations in the expression of these proteins, especially under lipotoxic stress, can significantly impair muscle regeneration and contribute to the progression of sarcopenia [7,8].

GLP-1 receptor agonists (GLP-1 RAs), such as liraglutide, have become widely used therapeutic agents for T2D in recent years due to their multiple benefits in managing blood glucose levels and supporting weight loss. Clinical studies have shown that liraglutide not only improves insulin sensitivity but also significantly reduces both body weight and body fat, representing an effective approach for improving the metabolic dysregulation seen in T2D [9]. However, despite its effectiveness, liraglutide has potential drawbacks. Although it facilitates weight loss, it can lead to muscle mass loss, particularly in patients already at risk of sarcopenia. This is concerning because sarcopenia worsens both metabolic and physical health outcomes in T2D patients [10]. This effect is exacerbated by elevated FFA levels in T2D, which lead to lipotoxicity, a condition characterized by the accumulation of excessive fat in muscle cells, thereby disrupting their function and regeneration [11]. To counteract the potential unfavorable effects of GLP-1 RAs on muscle mass, β-hydroxy-β-methylbutyrate (HMB) has gained attention as a potential muscle-preserving supplement. HMB is a metabolite of the branched-chain amino acid leucine, which plays a critical role in reducing muscle protein breakdown while promoting muscle protein synthesis. This dual action helps mitigate muscle loss in conditions of metabolic stress, such as T2D [12]. In particular, HMB has shown significant promise in combating sarcopenia in individuals with T2D, a population that is particularly vulnerable due to the combined effects of insulin resistance, chronic inflammation, and lipotoxicity [13]. By inhibiting the activity of catabolic pathways like the ubiquitin–proteasome system, which is often upregulated in response to metabolic stress, HMB can reduce the breakdown of muscle proteins and enhance muscle regeneration [14]. Moreover, HMB has been found to stimulate the expression of myogenic markers such as myogenin and MyoD, which are critical for muscle differentiation and regeneration [15]. In T2D patients, where muscle regeneration is impaired by factors like elevated FFAs and insulin resistance, HMB supports the restoration of normal muscle function by promoting muscle protein synthesis. Additionally, HMB can reduce the accumulation of reactive oxygen species (ROS) that is commonly seen in sarcopenia and metabolic diseases, which further contributes to muscle cell damage [16]. This antioxidant effect helps preserve muscle integrity by mitigating oxidative stress, a key player in the progression of sarcopenia in T2D.

However, whether HMB can serve as a muscle-preserving adjunct during liraglutide treatment under diabetic lipotoxic stress remains unknown. This question is clinically relevant because recent evidence has highlighted protein supplementation, resistance training, creatine, and HMB as potential strategies to support lean mass preservation during GLP-1 RA-associated weight loss [17]. Therefore, this study aimed to investigate the combined effects of liraglutide and HMB on lipotoxic and atrophic alterations in differentiated C2C12 myotubes exposed to high glucose and free fatty acids. The translational relevance of this study lies in its potential to support future combination strategies aimed at preserving skeletal muscle integrity while maintaining the metabolic benefits of GLP-1 receptor agonist therapy in patients vulnerable to sarcopenia.

## 2. Materials and Methods

### 2.1. Chemicals and Materials

Oleic acid (OA), palmitic acid (PA), 3-(4,5-dimethylthiazol-2-yl)-2,5-diphenyltetrazolium bromide (MTT), crystal violet, oil red-O, 4′,6-diamidino-2-phenylindole (DAPI), HMB, Calcein AM, JC-1, and 2′,7′-dichlorodihydrofluorescein diacetate (DCFH-DA) were purchased from Sigma-Aldrich (Munich, Germany). Rapamycin was obtained from Selleck Chemicals (Houston, TX, USA). 2-NBDG (2-Deoxy-2-[(7-nitro-2,1,3-benzoxadiazol-4-yl)amino]-D-glucose) was purchased from Invitrogen (Carlsbad, CA, USA). Liraglutide was purchased from Novo Nordisk (Bagsvaerd, Denmark). Primary antibodies against myogenin (sc-52903), MyoD (sc-32758), and MyHC (sc-376157) were obtained from Santa Cruz Biotechnology (Santa Cruz, CA, USA). Antibodies against pSer^307^-IRS1 (GTX133848), IRS1 (GTX31717), pSer^473^-Akt (GTX640148), Akt (GTX121937), atrogin-1 (GTX05209), and MuRF1 (GTX33334) were purchased from GeneTex (Irvine, CA, USA). Antibodies against pSer^9^-GSK3β (#9336) and GSK3β (#9315) were obtained from Cell Signaling Technology (Danvers, MA, USA). Antibodies against pSer^2448^-mTOR (AP0115) and mTOR (A11355) were purchased from ABclonal (Woburn, MA, USA). The antibody against β-actin (NB600-501) was obtained from Novus Biologicals (Littleton, CO, USA). FFAs, consisting of oleic acid and palmitic acid (OA:PA = 2:1), were prepared by dissolving the fatty acids in 0.1 M NaOH at 70 °C and subsequently conjugating them with 10% (*w*/*v*) bovine serum albumin (BSA) to obtain a stock solution, which was then diluted to the desired concentration for cell treatment as previously described [18].

### 2.2. Cell Culture and Viability Assay

The C2C12 mouse myoblast cell line (American Type Culture Collection, ATCC, Manassas, VA, USA) was cultured in Dulbecco’s Modified Eagle Medium (DMEM) supplemented with 10% fetal bovine serum (FBS) and 1% penicillin/streptomycin. Cells were maintained in a humidified incubator at 37 °C with 5% CO_2_. Prior to differentiation, the cells were passaged at a ratio of 1:3 for no more than 15 passages to ensure consistency and avoid senescence. Only cells between passages 5 and 15 were used for experimentation to maintain cellular integrity. For differentiation, cells were switched to differentiation medium containing 2% horse serum and incubated for 5 days to allow the formation of myotubes [19]. During culture, regular cell quality control was performed, including mycoplasma testing and verification of morphology using phase-contrast microscopy to ensure the absence of contamination and the presence of healthy cells. Cell viability was evaluated using the MTT assay. After treatment, 10 µL of MTT solution (5 mg/mL in phosphate-buffered saline, PBS) was added to the cells, and incubation continued for 4 h at 37 °C. The resulting formazan crystals were solubilized in 100 µL of dimethyl sulfoxide (DMSO). Absorbance was measured at 570 nm using the SpectraMax iD5 multi-mode microplate reader (Molecular Devices, San Jose, CA, USA). The absorbance values were normalized to the untreated control group, and cell viability was expressed as a percentage of the control.

### 2.3. Crystal Violet Staining

After treatment, C2C12 myotubes were washed twice with PBS to remove any residual media. The cells were then fixed in 4% paraformaldehyde at room temperature for 15 min. Following fixation, the cells were stained with a 0.5% crystal violet solution in methanol for 15 min to allow adequate staining of the cell structures. The cells were then washed thoroughly with distilled water to remove excess stain. The stained myotubes were examined under a light microscope to visually assess morphological changes.

### 2.4. Oil Red-O Staining

After treatment, C2C12 myotubes were washed twice with phosphate-buffered saline (PBS) to remove any residual medium. The cells were then fixed with 4% paraformaldehyde for 15 min at room temperature. Following fixation, the cells were washed with PBS and incubated with oil red-O solution (0.5% in isopropanol) for 15–20 min at room temperature to stain lipid droplets. After staining, the cells were thoroughly washed with PBS to remove excess dye. For quantitative analysis of intracellular lipid accumulation, the retained dye was eluted with isopropanol, and absorbance was measured at 510 nm using a SpectraMax iD5 multi-mode microplate reader (Molecular Devices, San Jose, CA, USA). The absorbance values were used to quantify the lipid content, with higher absorbance indicating greater lipid accumulation in the cells.

### 2.5. Lactate Dehydrogenase (LDH) Measurement

Lactate dehydrogenase (LDH) release was measured using the LDH assay kit (ab102526, Abcam, Cambridge, MA, USA). After treatment, the conditioned medium was collected, and the assay was performed according to the manufacturer’s protocol. Briefly, an aliquot of the medium was added to a 96-well plate, and the reaction mixture was prepared by adding the assay reagent. The conversion of the substrate to a formazan product was allowed to occur for a specified time, and absorbance was measured at 490 nm using a SpectraMax iD5 Multi-Mode Microplate Reader (Molecular Devices, Sunnyvale, CA, USA). Cytotoxicity was quantified by calculating the percentage of LDH release in the treated samples relative to the control group, using the following formula:

$$Cytotoxicity\left(\%\right)=\frac{Absorbance\left(treated\right)-Absorbance\left(background\right)}{Absorbance\left(\max control\right)-Absorbance\left(background\right)}\times 100$$

where max control represents the maximum LDH release obtained from cells subjected to complete lysis.

### 2.6. Nile Red Staining for Lipid Droplet and High-Content Analysis (HCA)

To assess lipid droplet accumulation, C2C12 myotubes were stained with Nile red according to the manufacturer’s protocol. After treatment, cells were washed twice with PBS and fixed with 4% paraformaldehyde for 15 min at room temperature. The cells were then stained with Nile red solution (1 µg/mL in PBS) for 30 min at 37 °C. After staining, the cells were washed twice with PBS to remove excess dye. DAPI was used for nuclear staining to aid in cellular localization. Lipid droplets were visualized using the ImageXpress micro confocal high-content imaging system (Molecular Devices, Sunnyvale, CA, USA). High-resolution images were acquired from at least 5 random fields of view to ensure comprehensive analysis. The MetaXpress software (ver. 6.7.0.211, Molecular Devices, Sunnyvale, CA, USA) was used for automated analysis of lipid droplet number, size, and distribution. The analysis specifically quantified lipid droplets based on their diameter, providing a detailed measure of lipid accumulation and distribution within the cells. DAPI staining was used to visualize cell nuclei for accurate localization and identification of myotubes.

### 2.7. Western Blot Analysis

Cells were lysed using Gold Lysis Buffer (50 mM Tris-HCl pH 7.4), 150 mM NaCl, 1% Triton X-100, 1 mM EDTA, 1 mM PMSF, 1 µg/mL aprotinin, 1 µg/mL leupeptin, and 1 mM sodium orthovanadate) supplemented with protease and phosphatase inhibitors (Thermo Fisher Scientific, Waltham, MA, USA) to prevent protein degradation. The protein concentration of the lysates was determined using the BCA protein assay kit (Bio-Rad, Hercules, CA, USA). Equal amounts of protein (30 μg) were separated by SDS-PAGE and transferred to polyvinylidene difluoride (PVDF) membranes (Millipore, Bedford, MA, USA) using a wet transfer method. After blocking with 5% bovine serum albumin (BSA) (Sigma-Aldrich, Munich, Germany) in PBST (phosphate-buffered saline with 0.1% Tween-20) for 1 h at room temperature, the membranes were incubated overnight at 4 °C with the appropriate primary antibodies, diluted in blocking solution. After washing with PBST three times, the membranes were incubated with horseradish peroxidase (HRP)-conjugated secondary antibodies for 1 h at room temperature. The protein bands were visualized using chemiluminescence with an enhanced chemiluminescence (ECL) detection kit (Cytiva, Marlborough, MA, USA) according to the manufacturer’s instructions. The intensity of the protein bands was quantified by densitometric analysis using ImageJ software (ver. 1.54). Data were normalized to the corresponding loading control (β-actin) to account for loading variations.

### 2.8. Calcein AM Staining

To assess myotube morphology and cell viability, C2C12 myotubes were stained with Calcein AM (1 μM), a fluorescent dye that indicates live cell viability. After treatment, the cells were incubated with Calcein AM for 30 min at 37 °C in a humidified incubator. Following incubation, the cells were washed twice with PBS to remove excess dye. The stained cells were visualized using a fluorescence microscope (CKX41 with DP74 camera, Olympus, Tokyo, Japan), with excitation/emission wavelengths set at 494/517 nm to observe the live cells and assess myotube morphology. The myotube area was quantified using ImageJ software (ver. 1.54) by measuring the Calcein AM-positive area as an index of myotube preservation.

### 2.9. 2-NBDG Uptake

To assess glucose uptake in C2C12 myotubes, cells were treated with 2-NBDG at a concentration of 100 µM for 30 min at 37 °C. After treatment, the cells were washed twice with PBS to remove excess dye. The uptake of 2-NBDG was visualized using a fluorescence microscope (CKX41 with DP74 camera, Olympus, Tokyo, Japan) with excitation/emission wavelengths set at 485/530 nm. The intensity of fluorescence was quantified using ImageJ software (ver. 1.54) to assess glucose uptake, with higher fluorescence indicating greater uptake of 2-NBDG into the myotubes.

### 2.10. Analysis of Mitochondrial Membrane Potential by JC-1

Mitochondrial membrane potential (ΔΨm) was assessed using JC-1 dye. After treatment, cells were incubated with JC-1 (2 μM) for 30 min at 37 °C. Following incubation, cells were washed twice with PBS to remove any excess dye. The stained cells were visualized using an Olympus CKX41 microscope equipped with a DP74 camera (Olympus, Tokyo, Japan). Fluorescence was captured using the following excitation/emission wavelengths: 490/530 nm for the JC-1 monomer (green fluorescence) and 525/590 nm for the JC-1 aggregate (red fluorescence). The red-to-green fluorescence ratio was used as an indicator of mitochondrial membrane potential. For quantification, the fluorescence images were analyzed using ImageJ software (ver. 1.54). The intensity of both red and green fluorescence was measured, and the red-to-green fluorescence ratio was calculated to assess changes in mitochondrial membrane potential.

### 2.11. Measurement of Reactive Oxygen Species (ROS)

ROS production was measured using the fluorescent probe DCFH-DA. After treatment, cells were incubated with DCFH-DA (10 μM) for 30 min at 37 °C. The probe was then converted to a fluorescent product (DCF) in the presence of ROS. After incubation, the cells were washed twice with PBS to remove excess probe and analyzed using a Novocyte flow cytometer (ACEA Biosciences, San Diego, CA, USA). ROS levels were assessed by measuring the fluorescence intensity of DCF. Higher fluorescence intensity indicates increased ROS production. For quantitative analysis, the flow cytometry data were processed using FlowJo software (ver. 10, TreeStar, Ashland, OR, USA) to analyze the ROS levels in the treated samples. The fluorescence intensity was normalized to the control group to assess ROS generation.

### 2.12. mRNA Expression Analysis by Reverse-Transcription Quantitative PCR (qPCR)

Total RNA was extracted from C2C12 cells using the RNeasy Kit (Qiagen, Germantown, MD, USA) according to the manufacturer’s protocol. RNA concentration and purity were assessed using a spectrophotometer to ensure RNA quality. For reverse transcription, 1 µg of total RNA was converted into complementary DNA (cDNA) using a TProfessional Thermocycler (Biometra, Göttingen, Germany) and a commercial reverse transcription kit, under optimized conditions. qPCR was performed using the Power SYBR Green PCR Master Mix (Applied Biosystems, Foster City, CA, USA) on the ABI 7300 Sequence Detection System. Each cDNA sample was analyzed in triplicate using the following cycling conditions: an initial denaturation step at 95 °C for 10 min, followed by 40 amplification cycles of 95 °C for 15 s and 60 °C for 1 min. A final dissociation curve analysis was performed to verify the specificity of the amplification. Relative mRNA expression levels for SOD1 (*Sod1*), SOD2 (*Sod2*), and catalase (*Cat*) were calculated using the 2^−ΔΔCt^ method and normalized to GAPDH as an internal control. The primers for the target genes and GAPDH (*Gapdh*) are listed in Table 1. Data analysis was performed using the Sequence Detection System software (ver. 2.4, Applied Biosystems, Foster City, CA, USA).

### 2.13. Immunocytochemistry Staining for MyHC

For immunocytochemical staining, C2C12 myotubes were fixed with 4% paraformaldehyde for 15 min at room temperature. After fixation, the cells were permeabilized with 0.1% Triton X-100 for 10 min to allow antibody access to intracellular targets. The cells were then incubated overnight at 4 °C with primary antibodies against MyHC (1:200 dilution), diluted in PBS containing 1% BSA (bovine serum albumin). After washing with PBS, the cells were incubated with the appropriate fluorescent secondary antibody (1:500 dilution) for 1 h at room temperature. To stain the nuclei, cells were incubated with DAPI (1 µg/mL) for 5 min at room temperature after secondary antibody incubation. After washing with PBS, the stained cells were visualized using the ImageXpress Micro Confocal High-Content Imaging System (Molecular Devices, Sunnyvale, CA, USA) to capture high-resolution images. The images were obtained from at least 5 random fields of view to ensure comprehensive analysis. The intensity of MyHC expression was quantified using MetaXpress software (ver. 6.7.0.211, Molecular Devices, Sunnyvale, CA, USA) to measure fluorescence intensity. The DAPI fluorescence was used to identify cell nuclei and to help localize the MyHC expression. The data were normalized to the background levels.

### 2.14. FOXO Transcriptional Activity Assay

FOXO (forkhead box O) transcriptional activity was evaluated using a FOXO reporter kit (#60643, BPS Bioscience, San Diego, CA, USA), which contains a firefly luciferase reporter driven by tandem FOXO-responsive elements and a constitutively expressed Renilla luciferase vector as an internal control. Differentiated C2C12 myotubes were transiently transfected with the FOXO reporter construct using Lipofectamine 3000 (Invitrogen, Carlsbad, CA, USA) according to the manufacturer’s instructions. Briefly, cells were seeded in 96-well plates and allowed to reach appropriate confluence prior to transfection. The reporter plasmid mixture, including the FOXO reporter and Renilla control plasmids, was prepared with Lipofectamine 3000 reagent and added to the cells, followed by incubation for 24 h to allow reporter expression. After transfection, cells were subjected to the indicated treatments. Following treatment, luciferase activities were measured using a dual-luciferase reporter assay system. Firefly and Renilla luminescence signals were sequentially detected using a SpectraMax iD5 multi-mode microplate reader (Molecular Devices, Sunnyvale, CA, USA). FOXO transcriptional activity was calculated as the ratio of firefly luciferase activity to Renilla luciferase activity to normalize for transfection efficiency.

### 2.15. Statistical Analysis

All quantitative data are presented as the mean ± standard deviation (SD) from at least three independent experiments. Statistical analyses were performed using SPSS software (ver. 25, SPSS Inc., Chicago, IL, USA). Comparisons among multiple groups were conducted using one-way analysis of variance (ANOVA), followed by Tukey’s post hoc test for pairwise comparisons when a significant overall difference was detected. A value of *p* < 0.05 was considered statistically significant.

## 3. Results

### 3.1. Establishment of an HG+FFA-Induced Lipotoxic but Sublethal Injury Model in Differentiated C2C12 Myotubes

**Figure 1:**
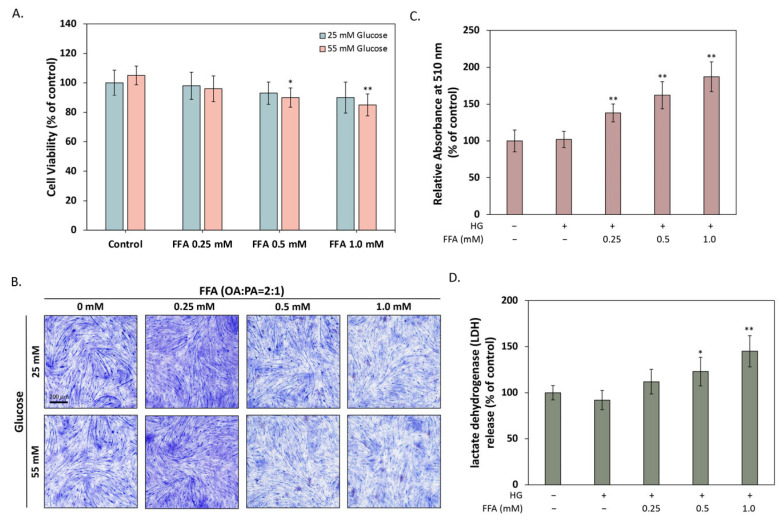
Differentiated C2C12 myotubes were exposed to 25 or 55 mM glucose in the presence of increasing concentrations of free fatty acids (FFAs; oleic acid: palmitic acid = 2:1) for 24 h to establish a diabetic lipotoxic injury model. (**A**) Cell viability was assessed by MTT assay. Treatment with 55 mM glucose plus 0.25 mM FFAs did not induce significant cytotoxicity, whereas FFA concentrations above 0.5 mM caused a mild reduction in cell viability. (**B**) Representative bright-field images of crystal violet staining showing the overall morphology of differentiated myotubes after treatment. Gross myotube morphology was largely preserved under most conditions, although slightly reduced staining intensity was observed at higher FFA concentrations. (**C**) Quantification of oil red-O staining after dye extraction with isopropanol. Intracellular lipid accumulation was significantly increased from 0.25 mM FFAs onward. (**D**) Lactate dehydrogenase (LDH) levels in conditioned medium were measured as an indicator of cellular injury. LDH release was significantly increased at FFA concentrations of 0.5 mM and above, indicating early cellular injury at higher FFA concentrations. Data are presented as mean ± SD from three independent experiments. * *p* < 0.05 and ** *p* < 0.01 compared with control.

To develop an *in vitro* model simulating lipotoxic stress under diabetic conditions while avoiding overt myotube damage, differentiated C2C12 cells were exposed to either 25 or 55 mM glucose in combination with increasing concentrations of FFAs (OA:PA = 2:1) for 24 h. As shown in Figure 1A, MTT analysis demonstrated that treatment with 55 mM glucose plus 0.25 mM FFAs did not significantly reduce cell viability, whereas FFA concentrations above 0.5 mM produced a mild cytotoxic effect. Consistent with this finding, crystal violet staining revealed that the overall morphology of differentiated myotubes remained largely preserved under most conditions, although a slight reduction in staining intensity was observed when FFAs exceeded 0.5 mM (Figure 1B). In contrast, quantitative analysis of oil red-O staining demonstrated that intracellular lipid accumulation was already significantly increased at 0.25 mM FFAs under high-glucose conditions and further increased with higher FFA concentrations (Figure 1C). In addition, LDH measurement in conditioned medium indicated a significantly increased cellular injury beginning at 0.5 mM FFAs compared with the control group (Figure 1D). Taken together, these results indicate that treatment with 55 mM glucose plus 0.25 mM FFAs is sufficient to induce marked intracellular lipid accumulation while avoiding substantial cytotoxicity or overt structural damage to differentiated myotubes. Therefore, this condition was selected as the pathological stimulus for subsequent experiments to model sublethal diabetic-like lipotoxic stress in differentiated myotubes.

### 3.2. Liraglutide Reduces Large Lipid Droplet Accumulation but Fails to Preserve the Myotube Phenotype Under HG+FFA Conditions

**Figure 2:**
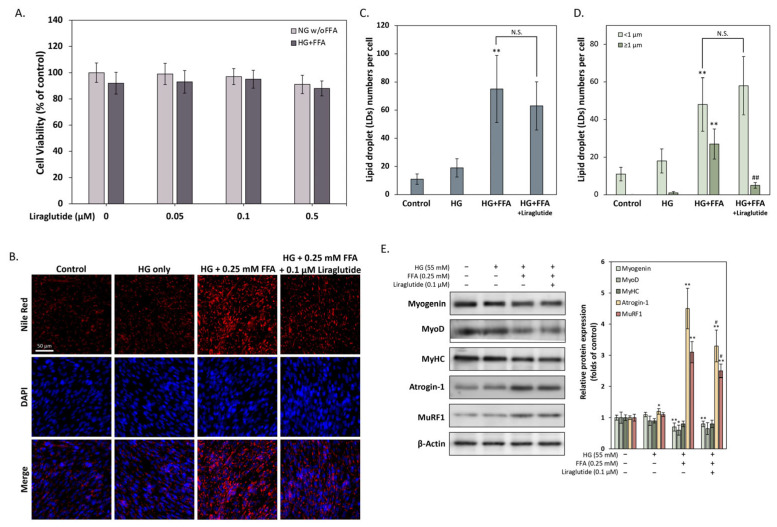
Differentiated C2C12 myotubes were used to evaluate the effects of the GLP-1 receptor agonist liraglutide under HG+FFA conditions. (**A**) Cell viability was assessed by MTT assay after treatment with liraglutide (0.05, 0.1, or 0.5 μM) for 24 h under normal or HG+FFA conditions. No significant reduction in cell viability was observed at any tested concentration. A 0.1 μM quantity of liraglutide was selected for subsequent experiments as a non-cytotoxic intermediate concentration. (**B**) Representative fluorescence images of Nile red staining showing intracellular lipid droplets (LDs) in differentiated C2C12 myotubes. Nile Red fluorescence indicates intracellular LDs, DAPI indicates nuclei, and merged images show the spatial distribution of LDs relative to nuclei. High glucose alone induced only minimal lipid accumulation, whereas HG+FFA markedly increased LD formation after 24 h. Co-treatment with 0.1 μM liraglutide visually attenuated this effect. (**C**) High-content analysis (HCA) of Nile red-stained cells showing the number of LDs per cell. Liraglutide did not significantly reduce the total number of LDs induced by HG+FFA. (**D**) HCA-based size distribution analysis of LDs. LDs were categorized by diameter into <1 μm and ≥1 μm groups. Liraglutide significantly reduced the number of larger LDs (≥1 μm), while showing little effect on smaller LDs. (**E**) Western blot analysis of myogenic markers (myogenin, MyoD, and MyHC) and atrophy-related proteins (atrogin-1 and MuRF1). HG+FFA slightly reduced the expression of myogenic proteins and markedly increased atrogin-1 and MuRF1 levels, indicating a mild disruption of mature myotube phenotype together with activation of an atrophic/catabolic program. Co-treatment with 0.1 μM liraglutide produced little improvement in these protein changes. Data are presented as mean ± SD from three independent experiments. * *p* < 0.05 and ** *p* < 0.01 compared with control; # *p* < 0.05 and ## *p* < 0.01 compared with HG+FFA-treated group.

To evaluate the effects of liraglutide on lipotoxic injury in differentiated C2C12 myotubes, cells were treated with liraglutide at 0.05, 0.1, or 0.5 μM for 24 h under either normal or HG+FFA conditions. MTT analysis showed that liraglutide did not significantly reduce cell viability at any tested concentration in either condition when compared with the control group (Figure 2A). Although no significant difference was observed among liraglutide-treated groups under HG+FFA stimulation, 0.1 μM liraglutide was selected for subsequent experiments as an intermediate non-cytotoxic concentration. Nile red staining revealed that high glucose alone induced only minimal intracellular lipid droplet accumulation, whereas HG+FFA treatment for 24 h markedly increased lipid droplet formation in differentiated C2C12 cells; this effect was visually attenuated by co-treatment with 0.1 μM liraglutide (Figure 2B). However, high-content analysis showed that liraglutide did not significantly reduce the total number of lipid droplets per cell under HG+FFA conditions (Figure 2C). When lipid droplets were further classified by size, liraglutide significantly decreased the number of larger lipid droplets (diameter ≥ 1 μm), while having little effect on smaller droplets (<1 μm) (Figure 2D). Western blot analysis further demonstrated that HG+FFA slightly reduced the expression of myogenic markers, including myogenin, MyoD, and MyHC, suggesting a modest disruption of the mature myotube phenotype, while markedly increasing the muscle atrophy-related proteins atrogin-1 and MuRF1, indicating a shift toward an atrophic/catabolic state (Figure 2E). Notably, co-treatment with 0.1 μM liraglutide produced little improvement in these myogenic or atrophy-associated factors, suggesting that although liraglutide partially alleviates lipid droplet enlargement, it is insufficient to prevent the loss of myotube phenotype under HG+FFA-induced diabetic-like stress.

### 3.3. HMB Preserves Myotube Area but Shows Limited Direct Effects on Lipid Droplet Accumulation Under HG+FFA Conditions

**Figure 3:**
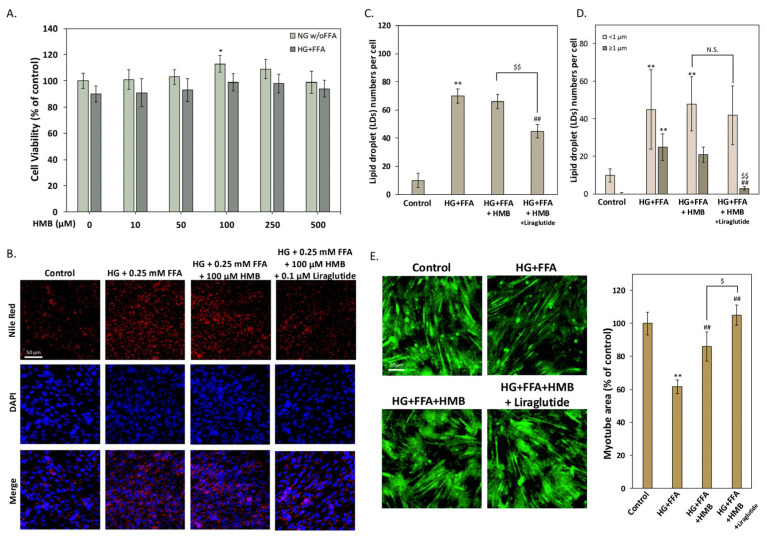
Differentiated C2C12 myotubes were used to evaluate the effects of β-hydroxy-β-methylbutyrate (HMB) under HG+FFA conditions. (**A**) Cell viability was assessed by MTT assay after treatment with HMB (10–500 μM) for 24 h under normal or HG+FFA conditions. No significant reduction in cell viability was observed at any tested concentration. A 100 μM quantity of HMB was selected for subsequent experiments as a non-cytotoxic intermediate concentration. (**B**) Representative fluorescence images of Nile red staining showing intracellular LDs in differentiated C2C12 myotubes. Nile red fluorescence indicates intracellular LD accumulation, DAPI indicates nuclei, and merged images show LD distribution relative to nuclei. HG+FFA markedly increased LD accumulation after 24 h. Treatment with 100 μM HMB alone had little visible effect on LD accumulation, whereas co-treatment with HMB and liraglutide clearly reduced LD formation. (**C**) HCA of Nile red-stained cells showing the number of LDs per cell. HMB alone did not significantly reduce HG+FFA-induced LD accumulation, while co-treatment with HMB and liraglutide significantly decreased the number of LDs per cell. (**D**) HCA-based size distribution analysis of LDs. LDs were categorized by diameter into <1 μm and ≥1 μm groups. HMB alone did not significantly alter the number of larger LDs (≥1 μm), whereas combined treatment with HMB and liraglutide significantly reduced this LD population. (**E**) Representative fluorescence images of Calcein AM staining showing live myotube morphology and area. HG+FFA markedly reduced the green fluorescent area, indicating loss of myotube area, whereas HMB treatment restored myotube preservation. This protective effect was further enhanced by co-treatment with HMB and liraglutide. Data are presented as mean ± SD from three independent experiments. * *p* < 0.05 and ** *p* < 0.01 compared with control; ## *p* < 0.01 compared with HG+FFA-treated group; $ *p* < 0.05 and $$ *p* < 0.01 compared with HG+FFA+HMB-treated group.

To determine whether HMB exerts protective effects against HG+FFA-induced myotube injury, differentiated C2C12 cells were treated with HMB at concentrations ranging from 10 to 500 μM for 24 h under either normal or HG+FFA conditions. MTT analysis showed that HMB did not significantly reduce cell viability at any tested concentration in either condition when compared with the control group (Figure 3A). Although no statistically significant differences were observed among HMB-treated groups under HG+FFA stimulation, 100 μM HMB was selected for subsequent experiments as an intermediate non-cytotoxic concentration. Nile red staining demonstrated that HG+FFA markedly increased intracellular lipid droplet accumulation after 24 h, whereas treatment with 100 μM HMB alone produced little obvious effect on this lipotoxic phenotype (Figure 3B). In contrast, co-treatment with HMB and liraglutide visibly reduced HG+FFA-induced lipid droplet accumulation. Consistent with the fluorescence images, high-content analysis revealed that HMB alone did not significantly decrease the total number of lipid droplets per cell under HG+FFA conditions, while the combination of HMB and liraglutide significantly reduced lipid droplet number (Figure 3C). Further size-based analysis showed that HMB alone did not significantly alter the number of larger lipid droplets (diameter ≥ 1 μm), whereas co-treatment with HMB and liraglutide markedly reduced this population (Figure 3D). To assess myotube preservation, Calcein AM staining was performed to visualize live myotube morphology. HG+FFA treatment markedly decreased the green fluorescent area, indicating substantial loss of myotube area, whereas HMB treatment clearly reversed this reduction. Notably, combined treatment with HMB and liraglutide further improved myotube preservation compared with HMB alone (Figure 3E). Together, these findings suggest that HMB alone has limited direct effects on HG+FFA-induced lipid droplet accumulation but exerts a clear protective effect on myotube maintenance, and this effect is further enhanced when combined with liraglutide.

### 3.4. Combined HMB and Liraglutide Treatment Restores Insulin Signaling, Glucose Uptake, Mitochondrial Function, and Redox Homeostasis Under HG+FFA Conditions

**Figure 4:**
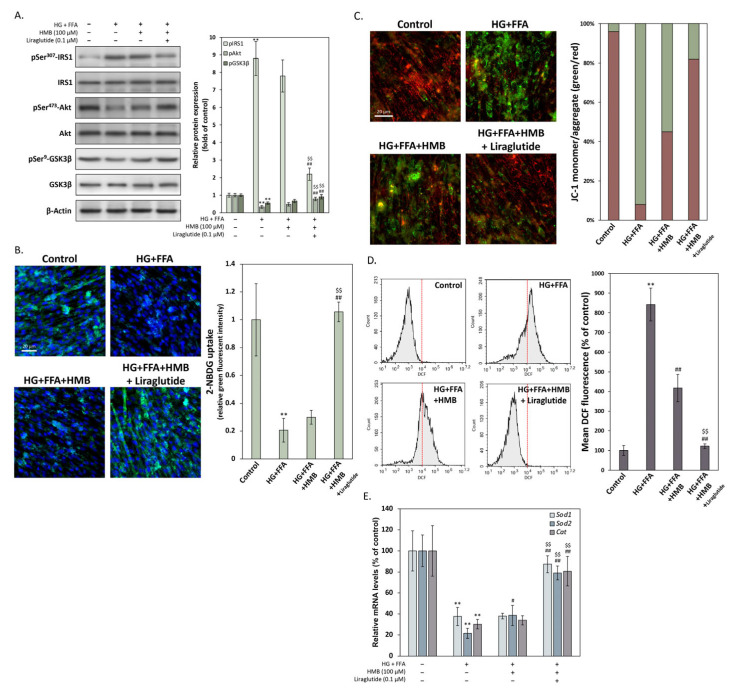
Differentiated C2C12 myotubes were used to examine the combined effects of HMB and liraglutide on HG+FFA-induced metabolic dysfunction. (**A**) Western blot analysis of insulin signaling-related proteins. HG+FFA increased the inhibitory phosphorylation of IRS1 at Ser307 and reduced Akt phosphorylation at Ser473, indicating blockade of insulin signaling; this was accompanied by decreased pSer^9^-GSK3β, consistent with impaired downstream Akt signaling. HMB alone produced little obvious improvement in these changes, whereas combined treatment with HMB and liraglutide reversed the HG+FFA-induced insulin signaling impairment. (**B**) Glucose uptake was assessed using the fluorescent glucose analog 2-NBDG. HG+FFA markedly suppressed glucose uptake in differentiated C2C12 myotubes, while HMB alone had little effect. In contrast, combined treatment with HMB and liraglutide significantly restored glucose uptake under HG+FFA conditions. (**C**) Mitochondrial membrane potential (ΔΨm) was evaluated by JC-1 staining. HG+FFA reduced red fluorescence (JC-1 aggregates) and increased green fluorescence (JC-1 monomers), indicating mitochondrial depolarization. HMB alone partially attenuated the reduction in ΔΨm, whereas the combination of HMB and liraglutide more effectively restored mitochondrial membrane potential. (**D**) Intracellular reactive oxygen species (ROS) were detected by DCFH-DA staining and quantified by flow cytometry. HG+FFA markedly increased ROS accumulation, HMB alone modestly attenuated this effect, and combined HMB plus liraglutide treatment significantly reduced HG+FFA-induced ROS generation. (**E**) Quantitative PCR analysis of antioxidant defense-related genes. HG+FFA significantly reduced the mRNA expression of *Sod1*, *Sod2*, and *Cat*. HMB alone showed limited effects on restoring these transcripts, whereas co-treatment with HMB and liraglutide significantly recovered their expression. Data are presented as mean ± SD from three independent experiments. ** *p* < 0.01 compared with control; # *p* < 0.05 and ## *p* < 0.01 compared with HG+FFA-treated group; $$ *p* < 0.01 compared with HG+FFA+HMB-treated group.

To further determine whether combined treatment with HMB and liraglutide provides complementary protection against HG+FFA-induced metabolic stress in differentiated C2C12 myotubes, insulin signaling, glucose uptake, mitochondrial membrane potential, intracellular ROS accumulation, and antioxidant gene expression were evaluated. Western blot analysis showed that HG+FFA markedly increased the inhibitory phosphorylation of IRS1 at Ser307 while reducing Akt phosphorylation at Ser473, indicating impaired insulin signaling; this was further supported by a reduction in pSer^9^-GSK3β, a downstream readout of Akt signaling (Figure 4A). HMB alone had little obvious effect on these HG+FFA-induced changes, whereas combined treatment with HMB and liraglutide clearly reversed the blockade of insulin signaling. Consistent with these findings, 2-NBDG uptake analysis demonstrated that HG+FFA significantly suppressed glucose uptake in C2C12 myotubes, confirming the presence of insulin resistance-like impairment; HMB alone did not meaningfully restore glucose uptake, but the addition of liraglutide markedly increased glucose uptake under HG+FFA conditions (Figure 4B). JC-1 staining further showed that HG+FFA caused a marked loss of mitochondrial membrane potential, as evidenced by decreased red fluorescence (JC-1 aggregates) and increased green fluorescence (JC-1 monomers), indicating mitochondrial depolarization (Figure 4C). HMB alone partially attenuated this reduction in ΔΨm, whereas the combination of HMB and liraglutide more effectively restored mitochondrial membrane potential. Likewise, DCFH-DA staining followed by flow cytometric quantification revealed that HG+FFA markedly increased intracellular ROS levels; HMB alone provided only modest attenuation of ROS accumulation, while combined HMB and liraglutide treatment significantly suppressed HG+FFA-induced oxidative stress (Figure 4D). In agreement with these observations, qPCR analysis showed that HG+FFA substantially downregulated the mRNA expression of antioxidant defense-related genes, including *Sod1*, *Sod2*, and *Cat*, whereas HMB alone had little effect on restoring their expression. In contrast, co-treatment with HMB and liraglutide significantly recovered the mRNA levels of these antioxidant genes (Figure 4E). Collectively, these results indicate that although HMB alone exerts limited effects on insulin signaling and glucose uptake, the combination of HMB and liraglutide effectively alleviates HG+FFA-induced metabolic dysfunction, mitochondrial impairment, and oxidative stress in differentiated C2C12 myotubes.

### 3.5. Combined HMB and Liraglutide Treatment Preserves the Myotube Phenotype and Suppresses Atrophic Signaling in a Manner That Is at Least Partly Dependent on mTOR Signaling

**Figure 5:**
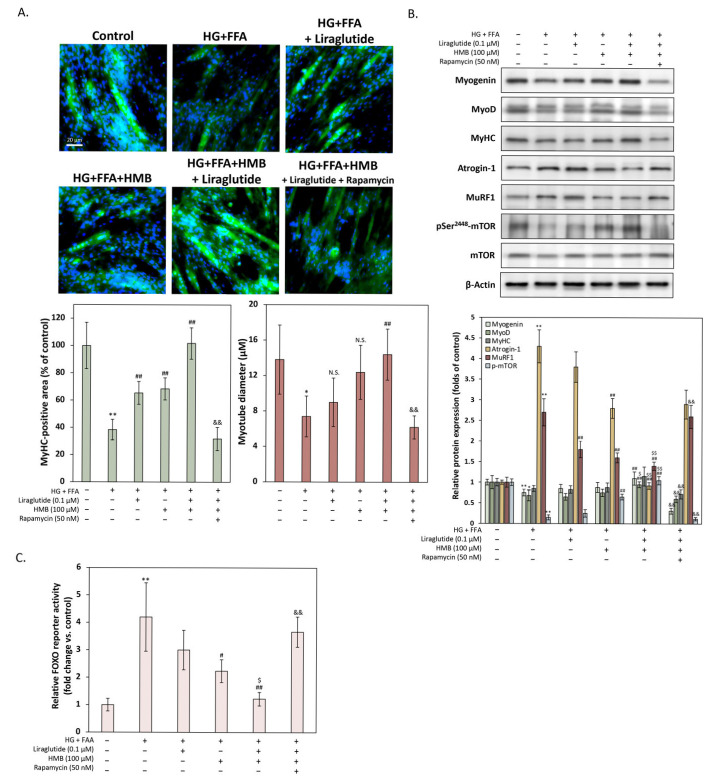
Differentiated C2C12 myotubes were used to determine whether combined treatment with HMB and liraglutide preserves myogenic phenotype and suppresses atrophic signaling under HG+FFA conditions. (**A**) Representative immunocytochemical images of MyHC staining (FITC, green) with nuclear counterstaining, together with high-content analysis of MyHC-positive area and myotube diameter. HG+FFA markedly reduced MyHC fluorescence intensity, MyHC-positive area, and myotube diameter. Treatment with HMB or liraglutide alone partially restored these parameters, whereas combined HMB and liraglutide treatment produced the strongest recovery. The addition of rapamycin (50 nM) largely abolished the protective effects of combined treatment. (**B**) Western blot analysis of myogenic markers (myogenin, MyoD, and MyHC) and atrophy-related proteins (atrogin-1 and MuRF1). HG+FFA slightly reduced myogenic protein expression while markedly increasing atrogin-1 and MuRF1 levels, indicating a shift toward an atrophic/catabolic state. HMB or liraglutide alone showed limited improvement, whereas combined HMB and liraglutide treatment significantly restored myogenic marker expression and suppressed atrophy-related proteins. These beneficial effects were attenuated by rapamycin. (**C**) FOXO transcriptional activity assay. HG+FFA significantly increased FOXO reporter activity compared with the control group, indicating activation of catabolic transcriptional signaling. HMB, but not liraglutide alone, partially suppressed this activation, whereas combined HMB and liraglutide treatment produced the greatest inhibitory effect. Rapamycin reversed the suppressive effect of combined treatment on FOXO reporter activity. Data are presented as mean ± SD from three independent experiments. * *p* < 0.05 and ** *p* < 0.01 compared with control; # *p* < 0.05 and ## *p* < 0.01 compared with HG+FFA-treated group; $ *p* < 0.05 and $$ *p* < 0.01 compared with HG+FFA+HMB-treated group; && *p* < 0.01 compared with HG+FFA+liraglutide+HMB-treated group.

To further determine whether combined treatment with HMB and liraglutide preserves myotube integrity under HG+FFA-induced diabetic-like stress, immunocytochemical, biochemical, and transcriptional analyses were performed. Immunocytochemical staining for the muscle-specific structural protein MyHC showed that HG+FFA markedly reduced MyHC fluorescence intensity, whereas treatment with HMB or liraglutide alone partially restored MyHC expression; notably, co-treatment with HMB and liraglutide produced the most prominent recovery of MyHC staining, while the addition of rapamycin (50 nM) largely abolished this protective effect (Figure 5A). Consistent with these observations, high-content analysis further demonstrated that HG+FFA significantly decreased the MyHC-positive fluorescent area and reduced myotube diameter, both of which were partially restored by HMB or liraglutide alone and more effectively recovered by combined HMB and liraglutide treatment; again, these beneficial effects were attenuated by rapamycin. Western blot analysis showed that HG+FFA slightly reduced the expression of the myogenic proteins myogenin, MyoD, and MyHC, while markedly increasing the muscle atrophy-related markers atrogin-1 and MuRF1, indicating a shift toward an atrophic/catabolic state (Figure 5B). Although HMB or liraglutide alone exerted only modest effects on these protein changes, their combination significantly restored myogenin, MyoD, and MyHC expression and suppressed atrogin-1 and MuRF1 levels, suggesting effective preservation of the myotube phenotype under HG+FFA conditions; importantly, these effects were largely reversed by rapamycin. Because FOXO transcription factors are key upstream regulators of atrophy-related genes, FOXO transcriptional activity was further assessed using a FOXO-responsive luciferase reporter assay. As shown in Figure 5C, HG+FFA treatment significantly increased FOXO transcriptional activity, indicating activation of catabolic signaling under lipotoxic stress. This elevation was partially attenuated by HMB treatment, whereas liraglutide alone showed a limited effect. Notably, combined treatment with HMB and liraglutide markedly suppressed FOXO activity, suggesting a combined inhibitory effect on catabolic transcriptional regulation. Importantly, this suppressive effect was largely reversed by rapamycin, indicating that the modulation of FOXO activity is, at least in part, dependent on mTOR signaling.

## 4. Discussion

In this study, we investigated the combined effects of the GLP-1 receptor agonist liraglutide and the nutritional metabolite HMB on preserving muscle integrity under a lipotoxic, insulin-resistant environment that models T2D-associated sarcopenia. Our findings demonstrate that while liraglutide alone can alleviate intracellular lipid accumulation and improve insulin signaling, it fails to robustly preserve the myogenic phenotype and muscle integrity in differentiated C2C12 myotubes exposed to HG+FFA. In contrast, HMB exhibits protective effects on myotube morphology and muscle differentiation markers, and when combined with liraglutide, we observe enhanced preservation of muscle structural and functional markers. These effects are accompanied by improved metabolic signaling and reduced oxidative stress. Collectively, our data provide foundational evidence for a novel therapeutic strategy to mitigate sarcopenia in the context of T2D. Although GLP-1 receptor agonists such as liraglutide have gained significant attention for their efficacy in improving glycemic control and promoting weight loss in T2D, their effects on muscle health remain underexplored. The muscle-wasting side effects of liraglutide have been noted in several reports, though their clinical relevance has not been fully investigated, especially in patients with sarcopenia, a condition prevalent in diabetic populations [20]. Existing studies have primarily focused on the metabolic benefits of liraglutide in controlling blood glucose and reducing fat mass, with limited attention paid to its potential to cause muscle loss. Given that sarcopenia exacerbates both insulin resistance and functional decline, it is crucial to address the negative effects of liraglutide on muscle mass in high-risk patients, particularly those with pre-existing muscle degradation. To our knowledge, our findings provide early evidence supporting HMB supplementation as a potential solution to mitigate muscle loss during liraglutide therapy.

HMB has garnered significant attention in recent years due to its practical application as a muscle-preserving supplement in various contexts such as nutritional stress, disuse atrophy, and oxidative damage. Studies have demonstrated that HMB supplementation is both safe and effective in promoting muscle health in populations at risk of muscle wasting, including older adults, individuals with chronic conditions, and athletes undergoing intense training [21]. Importantly, HMB has been shown to enhance muscle protein synthesis through the mTOR signaling pathway while inhibiting muscle protein degradation by suppressing the ubiquitin–proteasome system, a key pathway in catabolic processes [22]. This inhibition of catabolic signaling is critical, as muscle protein degradation is accelerated under conditions of chronic metabolic stress such as T2D [23]. HMB also reduces ROS and sustains mitochondrial function, both of which are critical in the pathophysiology of muscle degeneration under oxidative stress [24]. These properties highlight HMB’s versatility and safety profile, making it an attractive adjunctive therapy in clinical settings. Furthermore, HMB has been shown to modulate key anti-catabolic factors in muscle cells, including suppressing the activity of atrophy markers like atrogin-1 and MuRF1, which contribute to muscle wasting under metabolic stress [15]. In T2D-induced sarcopenia, where lipotoxicity from elevated FFAs exacerbates muscle wasting, our study demonstrates that HMB supplementation significantly reduces muscle degradation under lipotoxic stress. HMB also helps preserve myogenic markers (e.g., myogenin, MyoD, MyHC) in C2C12 myotubes exposed to HG+FFA, suggesting it sustains muscle integrity even under metabolic stress [15]. Recent studies further support HMB’s potential in counteracting muscle loss in diabetes and sarcopenia models, highlighting its efficacy in maintaining muscle mass and function in catabolic conditions [25].

On the other hand, liraglutide primarily acts through its glucose-lowering effects and its ability to promote weight loss. It achieves this by enhancing insulin secretion, improving insulin sensitivity, and reducing glucagon levels [26]. However, while liraglutide is effective in managing hyperglycemia and promoting fat loss, it is associated with muscle loss, especially in sarcopenic T2D patients. This muscle loss, likely due to both direct inhibition of muscle protein synthesis and indirect promotion of muscle atrophy through elevated catabolic signaling, presents a significant challenge when considering the long-term use of liraglutide in populations at risk for sarcopenia [27]. Our study suggests that HMB and liraglutide, when used together, can complement each other by targeting different mechanisms of muscle preservation and metabolic regulation. While liraglutide primarily addresses hyperglycemia and insulin resistance, HMB directly preserves muscle mass by inhibiting catabolic pathways and enhancing muscle protein synthesis. Together, they may provide complementary benefits by improving metabolic stress while preserving myotube integrity. Notably, liraglutide’s effect on glucose regulation combined with HMB’s anti-catabolic properties provides a multifaceted approach to treating sarcopenic T2D, an area that is currently underexplored in clinical research. However, clinical studies have already indicated that adequate protein supplementation can mitigate the risk of muscle loss associated with GLP-1 RA therapy [28], a perspective that aligns closely with our current findings. Moreover, while liraglutide has shown efficacy in reducing fat mass, which can potentially reduce muscle damage [29], it lacks direct muscle-protecting effects, which HMB provides. In our study, we observed that liraglutide could slightly reduce lipid accumulation but did not significantly improve muscle differentiation or regeneration markers in HG+FFA-treated myotubes. In contrast, HMB supplementation in the same conditions improved muscle health, supported myotube formation, and reduced oxidative stress, indicating that HMB’s protective effects are complementary to the benefits of liraglutide.

Mechanistically, a simplified interpretation of the present findings is that liraglutide and HMB exert complementary but convergent protective effects under HG+FFA-induced stress. Liraglutide appeared to act primarily on the metabolic component of injury by reducing lipid burden and improving insulin-related signaling, whereas HMB appeared to act more directly on the structural and anti-catabolic component by preserving myotube morphology and myogenic markers. When combined, these upstream effects may converge on the Akt/mTOR–FOXO regulatory axis, resulting in stronger preservation of the myotube phenotype and suppression of atrophy-related signaling. The finding that rapamycin largely attenuated these protective effects suggests that mTOR-associated signaling is a key downstream integration point required for the final muscle-preserving response. In this regulatory axis, HG+FFA impairs insulin-related anabolic signaling, thereby relieving the inhibitory constraint on FOXO-dependent catabolic transcription. In skeletal muscle, Akt functions as a central downstream effector of PI3K and suppresses atrophic signaling by phosphorylating FOXO transcription factors, which promotes their cytoplasmic retention and limits transcription of atrogenes such as atrogin-1 and MuRF1 [30]. In parallel, Akt positively regulates mTOR signaling to sustain protein synthesis and myogenic maintenance [31]. Thus, under lipotoxic and insulin-resistant conditions, reduced Akt activity is expected to simultaneously weaken mTOR-driven anabolic signaling and enhance FOXO-mediated catabolic transcription, thereby shifting muscle cells toward an atrophic state. Recent reviews have highlighted this coordinated PI3K/Akt/mTOR–FOXO network as a major regulatory hub governing skeletal muscle growth and atrophy under metabolic stress [2]. In this context, our data are highly consistent with the proposed pathway model. Combined HMB and liraglutide treatment restored Akt signaling, preserved myogenic markers, suppressed atrogin-1 and MuRF1 expression, and markedly reduced FOXO reporter activity, whereas rapamycin largely reversed these protective effects. These findings indicate that the complementary relationship between liraglutide and HMB should be understood as distinct upstream contributions that ultimately depend, at least in part, on a shared mTOR-associated anabolic pathway, rather than as two completely independent downstream mechanisms. Further studies are needed to clarify the precise interplay among Akt, mTOR, and FOXO in mediating these protective effects in diabetic muscle.

While this study provides valuable insights into the combination of liraglutide and HMB, there are several limitations to consider. First, the use of C2C12 myotube cells in this study, while informative, does not fully replicate the human muscle environment. *In vivo* studies using diabetic animal models and long-term clinical trials in T2D patients with sarcopenia are necessary to confirm the effectiveness of this combination therapy. In addition, obesity without overt T2D may represent a distinct metabolic condition in which skeletal muscle is exposed predominantly to lipotoxic stress without sustained glucotoxicity. Although the present study focused on T2D-associated glucolipotoxic stress using combined high-glucose and FFA exposure, future studies should evaluate the effects of GLP-1 receptor agonists with or without HMB under FFA-induced lipotoxic conditions alone. This approach would help determine whether HMB can also preserve skeletal muscle integrity during GLP-1 receptor agonist treatment in the context of obesity-associated sarcopenia independent of hyperglycemic stress. Second, while we observed that HMB improved muscle differentiation and preserved muscle markers, further investigation is needed to understand the dose–response relationship and the mechanisms by which HMB enhances muscle regeneration in the context of T2D. Third, while liraglutide’s effects on fat mass are well-documented, its long-term impact on muscle strength in sarcopenic patients remains to be explored. Further research should investigate whether other muscle-preserving agents combined with liraglutide can reduce the adverse effects on muscle mass. Additionally, another limitation of the present study is that the HG+FFA condition used here may better represent an acute and intensified metabolic/lipotoxic stress rather than the chronic, lower-grade metabolic derangement observed in clinical T2D. The use of 55 mM glucose plus 0.25 mM FFAs for 24 h was selected to establish a reproducible sublethal model that could induce robust lipid accumulation and early metabolic and atrophic alterations while avoiding substantial cytotoxicity or overt structural disruption of differentiated myotubes. Therefore, although this model is useful for evaluating early protective responses against diabetic-like lipotoxic stress, it does not fully recapitulate the long-term metabolic environment of T2D-associated sarcopenia. Future studies using lower glucose and FFA concentrations with longer exposure durations, together with chronic *in vivo* models and clinical investigations, will be needed to validate the translational relevance of these findings and to define appropriate dosing and treatment strategies. 

## 5. Conclusions

In conclusion, our study highlights a potential combined approach to managing T2D-associated sarcopenia by combining liraglutide and HMB. While liraglutide effectively addresses glycemic control, HMB offers an essential complementary role in preserving muscle integrity, reducing muscle wasting, and mitigating lipotoxic effects. This combination may represent a promising therapeutic strategy for T2D-associated sarcopenia, although further validation in chronic *in vivo* and clinical settings is required.

## Figures and Tables

**Table 1:** Primer sequences used for qPCR analysis.

Genes	Forward (5′-3′)	Reverse (5′-3′)
*S* *od* *1*	AAGGACGGCGGTGTTTATGG	TCTCCAGGTCTCCCCAGTCA
*Sod2*	GTGGAACCTCACATCAACGC	TGTGTTCCTTCCAGGTTCAGT
*Cat*	GGAGATGGACACAGTGAAGC	GCACACAGTGGGTTCTTCTC
*Gapdh*	TGTGTCCGTCGTGGATCTGA	TTGCTGTTGAAGTCGCAGGAG

## Data Availability

The data presented in this study are available on reasonable request from the corresponding authors.

## Abbreviations

The following abbreviations are used in this manuscript:

DAPI	4′,6-diamidino-2-phenylindole
DCFH-DA	2′,7′-dichlorodihydrofluorescein diacetate
DMSO	dimethyl sulfoxide
FFA	free fatty acid
FOXO	forkhead box O
GLP-1 RA	GLP-1 receptor agonist
HCA	high-content analysis
HG	high glucose
HMB	β-hydroxy-β-methylbutyrate
LDH	lactate dehydrogenase
MTT	3-(4,5-dimethylthiazol-2-yl)-2,5-diphenyltetrazolium bromide
MuRF1	muscle ring finger-1
MyHC	myosin heavy chain
MyoD	myogenic differentiation factor
2-NBDG	2-Deoxy-2-[(7-nitro-2,1,3-benzoxadiazol-4-yl)amino]-D-glucose
OA	oleic acid
PA	palmitic acid
PBS	phosphate-buffered saline
ROS	reactive oxygen species
T2D	type 2 diabetes
